# Patient delay in TIA: a systematic review

**DOI:** 10.1007/s00415-018-8977-6

**Published:** 2018-07-19

**Authors:** L. Servaas Dolmans, Arno W. Hoes, Marie-Louise E. L. Bartelink, Niels C. T. Koenen, L. Jaap Kappelle, Frans H. Rutten

**Affiliations:** 1 Julius Center for Health Sciences and Primary Care, University Medical Center Utrecht, Utrecht University, Utrecht, The Netherlands; 2 Department of Neurology, University Medical Center Utrecht, Utrecht University, Utrecht, The Netherlands

**Keywords:** TIA, Minor stroke, Patient delay, Systematic review

## Abstract

**Background:**

Patients who suffer a transient ischemic attack (TIA) have a high short-term risk of developing ischemic stroke, notably within the first 48 h. Timely diagnosis and urgent preventive treatment substantially reduce this risk. We conducted a systemic review to quantify patient delay in patients with (suspected) TIA, and assess determinants related to such delay.

**Methods:**

A systematic review using MEDLINE and EMBASE databases up to March 2017 to identify studies reporting the time from onset of TIA symptoms to seeking medical help.

**Results:**

We identified nine studies providing data on patient delay, published between 2006 and 2016, with 7/9 studies originating from the United Kingdom (UK). In total 1103 time-defined TIA patients (no remaining symptoms > 24 h), and 896 patients with a minor stroke (i.e., mild remaining symptoms > 24 h) were included (49.1% men, mean age 72.2 years). Patient’s delay of more than 24 h was reported in 33.1–44.4% of TIA patients, with comparable proportions for minor stroke patients. Delays were on average shorter in patients interviewed at the emergency department than among patients seen at TIA outpatient clinics. Univariably associated with a shorter delay were (1) a longer duration of symptoms, (2) motor symptoms, (3) a higher ABCD2 score, and (4) correct patient’s recognition as possible ischemic cerebrovascular event.

**Conclusions:**

More than a third of patients experiencing a TIA delays medical attention for more than a day, thus critically extending the initiation of stroke preventive treatment. There still seems to be insufficient awareness among lay people that symptoms suggestive of TIA should be considered as an emergency. Additional data and multivariable analyses are needed to define main determinants of patient delay.

## Introduction

Symptoms of a transient ischemic attack (TIA) are typically short-lasting, often not very specific and can easily be misinterpreted or trivialized by both patients and physicians. Early recognition of TIA, however, is essential to enable a rapid start of stroke prevention, as the risk of a subsequent ischemic stroke is highest in the first days after the TIA [1, 2].

The EXPRESS study evaluated the effect of introducing a rapid access assessment by physicians of suspected TIA, and showed a reduction of median delay to first prescription of treatment from 20 days to 1 day, which led to an impressive decrease of 90-day recurrent stroke rate from 10.3 to 2.1% [3]. Similar low recurrence risks were reported in the SOS-TIA study evaluating the impact of a round-the-clock access clinic [4]. The introduction of rapid access TIA outpatient clinics since the beginning of this century has improved timely diagnostic assessment by neurologists, but also created a more common awareness among general practitioners that patients with symptoms suspected of TIA should be assessed and when diagnosed be treated immediately. Thus, the physician’s delay was reduced dramatically in the last decade. An important remaining challenge is the reduction of the patient’s delay.

In 2008, a systematic review was published on determinants of patient delay in seeking medical attention after TIA. However, just one study included only patients with TIA; the other eight studies included both patients with stroke and (a minority of) TIA patients [5]. Most (7/9) studies were performed in the emergency department (ED) among patients suspected of stroke (still symptomatic) within the scope of thrombolysis, and provided ‘prehospital delay’ without subdivision in patient’s delay, general practitioner’s delay, and transportation time. Thus, conclusions on patient delay in (suspected cases of) TIA could not be drawn from this review.

Patients suspected of TIA are distinct from patients suspected of stroke in that the duration of symptoms is shorter, symptoms are often milder, and by definition transient. This has a large impact on the interpretation of symptoms by patients, possible bystanders, but also physicians. Better knowledge of patient delay and its determinants within the specific domain of TIA could help improving public education to increase lay awareness.

We aimed to quantify patient delay and assess its determinants in patients (suspected of) TIA and performed a systematic review.

## Methods

We conducted a literature search following PRISMA guidelines, and using MEDLINE and EMBASE databases from 1966 to March 1, 2017 [6]. The key terms presented in Box 1 were used to identify papers evaluating patient delay in TIA patients. Alternative terms for ‘delay’ had no added value in the search strategy.


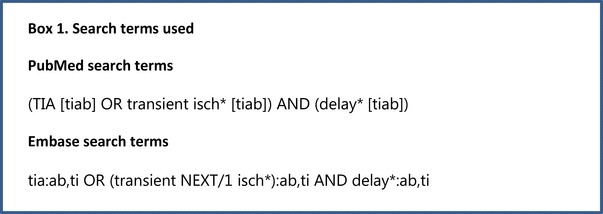


All abstracts were screened for relevance. We included primary studies assessing the time from onset of TIA symptoms to medical help-seeking. Since the domain of suspected TIA in daily practice also includes patients that are subsequently labeled with a diagnosis of minor stroke (i.e., mild remaining symptoms lasting longer than 24 h), studies reporting on both TIA and minor stroke were included in the review. If populations consisted of both major ischemic stroke and TIA patients, we only considered studies that provided separate data for TIA patients. We excluded articles in other languages than English or Dutch and conference abstracts. Full text versions of the potentially eligible studies were reviewed by two reviewers, and reference lists of all relevant articles were cross-checked for other relevant papers. Any disagreement was resolved by discussion.

Data were extracted using a standardized data extraction form, including an assessment of risk of bias (related to patient selection and the assessment of time delay and other variables) and applicability to our research objective. Next to data on delay to the first medical contact, we collected the results from analyses of possible determinants of such delay.

We considered studies that either used the ‘time-based’ or the ‘tissue-based’ definition of TIA. In both definitions the transiency of symptoms is the key characteristic distinguishing TIA from (minor) stroke. The time-based definition is based on a maximum duration of symptoms of 24 h, and the tissue-based definition on the absence of acute infarction with brain imaging [7]. We assessed the definitions distinguishing minor from major stroke handled in the original studies, since a uniform definition is lacking. Main differences concern the chosen value of the National Institutes of Health Stroke Score (NIHSS, a score ranging from 0 to 42 points that quantifies the severity of a stroke on different domains) to define minor stroke, usually ranging from ≤ 3 to ≤ 9.

Because of the heterogeneity of the data we did not aim to pool the data.

## Results

**Fig. 1:**
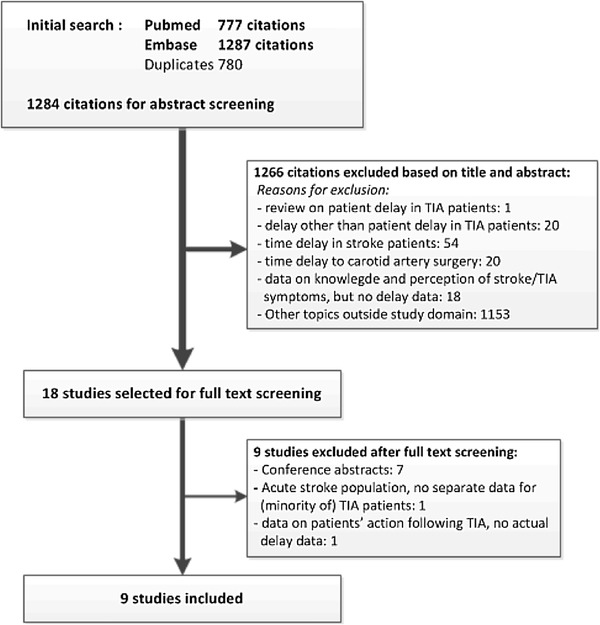
Flowchart of the literature review process

Our search yielded a total of 1284 studies. Figure 1 shows the flowchart of the review process. Eighteen studies could be selected for full text screening, and nine studies met our eligibility criteria. Table 1 gives an overview of the included studies, originating from the UK (*n* = 7), Spain (*n* = 1) and Norway (*n* = 1), and published between 2006 and 2016. Overall, taking into account overlap in study populations, these studies included 2657 participants with a cerebrovascular event. Delay data of 1103 (41.5%) TIA patients and 896 (33.7%) minor stroke patients were included in the analysis (49.1% men, mean age 72.2 years).

**Table 1:** Studies that assessed TIA patient’s delay

First author, year of publication	*n* (TIA/total)	Type of patients^a^	Setting	Delay TIA patients	Factors assessed
Studies using a quantitative approach for analysis of patient interviews					
Giles, 2006	241/241	TIA	Population-wide (OXVASC) and TIA clinics, UK	44.4% > 24 h	Clinical characteristics, patient’s perception, stroke risk
Rothwell, 2007	485/1278	TIA, MS	Population-wide (OXVASC), UK	41.8% > 24 h (TIA/MS)	–
Lasserson, 2008	359/793	TIA, MS	Population-wide (OXVASC), UK	Median time to contact GP: surgery hours 4.0 h, out of office hours 24.8 h (TIA/MS)	Time of onset of symptoms
Chandratheva, 2010	459/1000	TIA, MS	Population-wide (OXVASC), UK	TIA: 47.2% < 3 h, 33.1% > 24 h MS: 46.4% < 3 h, 26.1% > 24 h	Clinical characteristics, patient’s perception stroke risk
Geffner, 2012	70/388	TIA, MS, IS, ICH	Single ED, Spain	Median 0.5 h (IQR 0.25–1.50) (TIA only)	–
Faiz, 2013	100/440	TIA, MS, IS, ICH	Single ED/stroke unit, Norway	Median 2.0 h (IQR 0.5–12.8) (TIA only)	–
Wilson, 2014	222/278	TIA, MS	Single TIA clinic, UK	TIA: Median 3.5 h (IQR 0.5–41.5); MS: Median 6.0 h (IQR 0.5–25.8)	Clinical characteristics, patient’s perception, time of onset of symptoms
Hurst, 2016	103/150	TIA, MS	Single TIA clinic, UK	38.7% > 1 h 27.3% > 24 h (TIA/MS)	–
Study using a qualitative approach for analysis of patient interviews					
McSharry, 2014	20/20	TIA	Three TIA clinics, UK	60% < 1 h 20% > 24 h	Patient’s perception, role family/friends

*TIA* transient ischemic attack, *OXVASC* the Oxford Vascular Study, *UK* United Kingdom, *ED* emergency department, *MS* minor stroke, *IS* (major) ischemic stroke, *ICH* intracerebral hemorrhage, *IQR* interquartile range

^a^TIA was defined by the time-based definition in all studies

All included studies applied the time-based definition of TIA. Two studies restricted their study population to TIA patients only [8, 9]. Five studies also included minor stroke patients with an NIHSS ranging from less than 6 to less than 8 [3, 10–13]. Two studies included patients with (major) stroke, and only a small number of TIA patients. Both studies were executed in the ED setting [14, 15]. The other seven studies recruited patients from TIA outpatient clinics (*n* = 551) or were population-based (*n* = 1278). All studies included only cases that were confirmed to have TIA or minor stroke instead of suspected cases, and assessed the delay time after the diagnostic confirmation.

Four publications were generated by one single research group, including different (and overlapping) numbers of patients recruited over consecutive time periods and together constituting a cohort named the ‘Oxford Vascular study (OXVASC)’. This is a population-based collection of data of prospectively occurring acute vascular events in 91,000 adults registered at nine large group practices of general practitioners in Oxfordshire, UK.

The timing of the interview to assess patient’s delay was reported in four studies [8, 10, 13, 14], and ranged from up to 72 h after the onset of symptoms in the ED studies to a median of 22 days in the primary care OXVASC population.

### Delay

A summary of included studies with the data provided on delay is presented in Table 1. There is a large heterogeneity in the reporting of delays. Three studies provided data on TIA patients only, and recruited from TIA outpatient clinics or the population at large. Giles et al. (2006) and Chandratheva et al. (2010), both studies from the OXVASC group, showed that of the TIA patients (*n* = 241 and *n* = 459, respectively, with an overlap of 138 patients), 44.4 and 33.1% had a delay of more than 24 h [8, 10]. Wilson et al. (2014) reported a median delay of 3.5 (IQR 0.5–41.5) h in 222 TIA patients from a single British TIA outpatient clinic [11]. Chandratheva et al. only presented median delays for males and females separately: 4.0 (IQR 0.5–45.5) for men and 4.9 (IQR 0.8–48.9) h for women.

Three studies presented delays for TIA and minor stroke patients combined. Rothwell et al. (2007) provided delay data of all OXVASC patients referred to the EXPRESS clinic; 41.8% (247/591) had a delay of more than 24 h [3]. Lasserson et al. (2008) performed a subgroup analysis within largely the same (OXVASC) study population showing that the median time to calling a general practitioner (GP) during out of office hours is much longer than during working hours [24.8 (IQR 9.0–54.5) versus 4.0 (IQR 1.0–45.5) h] [12]. Hurst et al. (2016) evaluated delays of TIA and minor stroke patients (*n* = 150) in a single TIA outpatient clinic in Oxford (UK), and found that 38.7% (58/150) reacted by immediately seeking medical attention, while 27.3% (41/150) had a delay of more than 24 h [13].

The delays of TIA patients reported by Geffner et al. (2012) and Faiz et al. (2013) originate from a Spanish and Norwegian ED population, respectively. In the Spanish cohort, 70 TIA patients had a median delay of 0.5 (IQR 0.3–1.5) h, versus 1.0 (IQR 0.3–7.0) h in 318 stroke patients (of which 281 minor and major ischemic stroke and 37 intracerebral hemorrhage). The 100 TIA patients in the Norwegian cohort had a median delay of 2.0 (IQR 0.5–12.8) h, versus 1.9 (0.5–5.9) h in 290 minor and major ischemic stroke patients, and 0.5 (0.2–2.0) h in 50 intracerebral hemorrhage patients [14, 15].

### Determinants of patient delay

Three studies assessed potential determinants of delay using a quantitative approach (Table 2). Three different statistical methods were used, namely Chi square for comparing proportions, (presumably) Wilcoxon–Mann–Whitney for comparing delay times, and univariate Cox proportional hazards analysis. The largest study by Chandratheva et al. identified seven variables that were univariably associated with shorter delay: (1) the patient realizes the symptoms could be caused by a TIA, (2) presence of motor symptoms, (3) long persistence of symptoms, (4) a high ABCD2 score (a score for stroke risk prediction, including age, blood pressure, clinical features, duration and diabetes), (5) presence of speech symptoms, (6) a history of previous stroke and (7) a lower Mini Mental State Examination (MMSE) score [9]. The first four of these variables were also found to be associated with shorter delay in one of the other two studies [8, 11]. None of the studies performed multivariable analyses.

**Table 2:** Overview of determinants of patient’s delay in three studies that performed univariable analysis

Study	*n*	Statistical method	Variables that were evaluated	
Giles et al., 2006	241 (TIA)	*Not reported in Methods* Chi square for trend in proportions (for immediate, same day, next day and ≥ 2 days action)	Positive association (with shorter delay)	
			*Motor symptoms*	*p* trend 0.011
			*Duration of symptoms* ≥ 60 min	*p* trend 0.004
			*Higher ABCD2 score*	*p* trend 0.001
			No association with Age, sex, correct recognition as TIA, (brain) territory, blood pressure at clinic, a history of hypertension/diabetes/TIA/stroke/acute coronary syndrome/atrial fibrillation, smoking	
Chandratheva et al., 2010	459 (TIA)	*Not reported in Methods* Compared stratified medians, presumably using Wilcoxon–Mann–Whitney	Positive association with *Correct recognition as TIA* Yes: median 2.3 h (IQR 0.5–24.3) No: median 7.3 h (IQR 1.0–50.2)	*p* 0.005
			*Motor symptoms* Yes: median 1.6 h (IQR 0.3–20.1) No: median 16.0 h (IQR 1.4–66.5)	*p* < 0.001
			*Speech symptoms* Yes: median 2.2 h (IQR 0.5–22.5) No: median 11.5 h (IQR 1.0–59.5)	*p* < 0.001
			*Duration of symptoms* < 10 min: median 25.0 h (IQR 5.1–111.9) 10–59 min: median 4.1 h (IQR 0.7–48.0) ≥ 60 min: median 2.0 h (IQR 0.5–24.8)	*p* < 0.001
			*ABCD2 ≥ 5* Yes: median 1.8 h (IQR 0.5–18.0) No: median 15.3 h (IQR 1.0–63.1)	*p* < 0.001
			*Previous stroke* Yes: median 1.0 h (IQR 0.5–13.7) No: median 5.2 h (IQR 0.8–48.5)	*p* 0.006
			*Mini Mental State Examination* ≤ 24: median 2.0 h (IQR 0.3–25.1) > 24: median 4.4 h (IQR 0.8–48.7)	*p* 0.006
			No association with Age, sex, blood pressure at clinic, a history of hypertension/ diabetes/TIA/myocardial infarction/atrial fibrillation, educational level, social class	
Wilson et al., 2014	278 (TIA/MS)	Univariate Cox proportional hazards analysis	Positive association with *Correct recognition as possible TIA/stroke* Yes: median 2.0 h (IQR 0.5–48.0) No: median 6.5 h (IQR 0.3–22.2)	*p* 0.009
			No association with Age, sex, FAST, type of symptoms, duration, previous TIA/stroke, weekend presentation, before/during/after FAST campaign, lay input	

*TIA* transient ischemic attack, *ABCD2* prognostic score for early stroke risk prediction, including the items age, blood pressure, clinical symptoms, duration and diabetes, *FAST* Face Arm Speech Time, tool for the early recognition of stroke symptoms, *IQR* interquartile range, *MS* minor stroke

McSharry et al. (2014) explored possible determinants of patient delay in a qualitative manner using a semi-structured interview in 20 TIA patients from three British TIA clinics. Concerning recognition of symptoms they concluded that awareness of typical stroke symptoms could lead to urgent action when symptoms were more severe. On the other hand, if symptoms were not severe or vague, delay was longer. Seven of the 20 patients realized that a TIA could be the cause of their symptoms. Nevertheless, four of them decided to wait and see, because they considered the symptoms as not being serious or requiring immediate action. Importantly, often friends and family were involved in the decision making, and if this was the case, delays were often shorter. In 5/20 cases the decision to seek medical help was fully taken by a witness of the symptoms of the patient (four times family/friends, once a nurse), and medical services were contacted by them within 1 h. In 8/20 cases the decision to seek health care advice was made by the patient and their relatives together. In the remaining 7/20 cases the patient sought medical care on his own, and in these cases the longest delays were seen [9].

## Discussion

Our systematic review of nine studies shows that around 40% of TIA patients delays seeking medical attention for more than 24 h, and this was similar for patients that eventually showed to have a minor stroke. Three studies provided data on determinants of patient’s delay, and fast disappearance of symptoms, symptoms not being recognized as possible TIA, absence of motor symptoms and a lower ABCD2 score were associated with a longer delay.

We could only identify studies among patients with established TIA (or minor stroke). To the best of our knowledge, there are no studies that evaluated patients *suspected of* TIA, that is, the domain of the actual diagnostic dilemma. In view of the uncertainty around the diagnosis of TIA for both patient and clinician, also evidence on the delay of *all* suspected cases is important. In a substantial portion of patients with suspected symptoms, a clear and definite diagnosis can not be made by the neurologist even after multiple additional investigations. Including only those with established TIA may create a selection of the more typical cases, which is likely to bias (and most probably will underestimate) delay times and the determinants related to delay. Moreover, interviewing patients *after* they underwent additional investigations and were informed about their final diagnosis induces the risk of ‘recall bias’ and is likely to identify those symptoms typically known to be associated with established TIA.

Another important concern about the included studies is that those from the UK (notably Oxfordshire) were over-represented with also some patients reported in more than one manuscript. Therefore, some caution is warranted generalizing the results of this review, more because the organization and accessibility of care that can differ per region and country has an impact on patient delay.

Delays were on average much shorter in patients interviewed at the ED than those seen at TIA outpatient clinics, underlining the impact of the study setting on delay. The ED population must be regarded as a selection of patients that act rapidly to receive medical help, and very likely experience more ‘severe’ or typical symptoms. Surveys at TIA outpatient clinics provide a better reflection of patient delay in the complete spectrum of TIA patients presenting via different health care routes.

Bruins Slot et al. investigated the prehospital delay of patients with symptoms suspected of acute coronary syndrome (ACS) in the Dutch primary care setting, excluding patients who required instant hospital referral. The median patient delay was just 2.2 h, much shorter than the delays of the TIA patients in our review [16]. This is in line with the general opinion that symptoms such as chest pain and acute dyspnoea create much more sense of urgency in patients (but also in bystanders and relatives) than symptoms suggestive of neurological dysfunction. A general lack of knowledge about the need for urgency in the case of a TIA may account at least partly for this immense difference in delay between patients with suspected ACS symptoms and TIA symptoms.

The three studies that aimed to find determinants of patient delay quantitatively showed some similar results but also reported discrepancies. These studies used different questionnaires and applied different statistical methods, and per study relatively small numbers of participants (ranging from 241 to 459) were evaluated. None of the studies applied multivariable analyses, and it is therefore impossible to draw conclusions about which variables independently predict delay. Most likely, event characteristics like the type of symptoms do influence delay, but relations are more complex and interactions exist with other factors such as severity of symptoms. This may partly explain why some items are not identified in all studies.

The importance of *recognition* of symptoms remains heavily debated. The qualitative study by McSharry et al. may provide an explanation for the conflicting data on the role of recognition, stating that awareness of typical stroke symptoms may lead to action in case of more severe symptoms but may cause delay when symptoms are mild or vague. Furthermore, recognizing TIA symptoms is one thing, but many lay people are still unaware of the need for urgency, and, importantly, the fact that urgency remains even if symptoms disappear rapidly.

The most important limitation of this review is the heterogeneity between studies. The differences in study population and setting complicate the interpretation of the data. In addition, selective reporting in the original papers made it impossible to present delay times in a uniform way. Therefore, we reported what was available, e.g., either a median delay or delays categorized by a cut-off point, and we were not able to pool the findings.

Our review demonstrates that delay by patients frequently hampers a rapid start of treatment to prevent a subsequent stroke. As much as this poses a clinical problem, this also offers ample opportunity to implement measures to reduce delay time. Campaigns like Face Arm Speech Time (FAST) that educate on recognizing stroke symptoms are important examples of initiatives to gain time. The few data on the impact of the FAST campaign suggest a positive effect on awareness of stroke symptoms. However, the effect on patient’s response is limited, and thus it is emphasized that future campaigns should strengthen the response to stroke symptoms; the need to immediately respond and contact a health care professional [17, 18]. Lay people need to be better informed about the early risk of stroke and the need for an urgent call after a TIA, also when symptoms are short-lasting.

Additional data on patient’s perception and determinants of delay are needed. Given aforementioned considerations, we would like to recommend that future studies consider (1) including patients *suspected of* TIA, (2) conducting interviews before the eventual diagnosis is set by a neurologist, and (3) performing multivariable analyses to adequately weigh determinants of delay.

We conclude that too many patients with TIA delay seeking medical attention for a substantial time period, and thus risk a delay in receiving treatment to prevent subsequent stroke. More public education and attention for the symptoms of TIA are needed, stressing the importance of immediate action to prevent the occurrence of a stroke.

## References

[1] Giles MF, Rothwell PM (2007). Risk of stroke early after transient ischaemic attack: a systematic review and meta-analysis. Lancet Neurol.

[2] Wu CM, McLaughlin K, Lorenzetti DL (2007). Early risk of stroke after transient ischemic attack: a systematic review and meta-analysis. Arch Intern Med.

[3] Rothwell PM, Giles MF, Chandratheva A (2007). Effect of urgent treatment of transient ischaemic attack and minor stroke on early recurrent stroke (EXPRESS study): a prospective population-based sequential comparison. Lancet.

[4] Lavellée PC, Mesequer E, Abboud H (2007). A transient ischaemic attack clinic with round-the-clock access (SOS-TIA): feasibility and effects. Lancet Neurol.

[5] Sprigg N, Machili C, Otter ME (2009). A systematic review of delays in seeking medical attention after transient ischaemic attack. J Neurol Neurosurg Psychiatry.

[6] Liberati A, Altman DG, Tetzlaff J (2009). The PRISMA statement for reporting systematic reviews and meta-analyses of studies that evaluate health care interventions: explanation and elaboration. PLoS Med.

[7] Easton JD, Saver JL, Albers GW (2006). Definition and evaluation of transient ischemic attack: a scientific statement for healthcare professionals from the American Heart Association/American Stroke Association Stroke Council; Council on Cardiovascular Surgery and Anesthesia; Council on Cardiovascular Radiology and Intervention; Council on Cardiovascular Nursing; and the Interdisciplinary Council on Peripheral Vascular Disease. The American Academy of Neurology affirms the value of this statement as an educational tool for neurologists. Stroke.

[8] Giles MF, Flossman E, Rothwell PM. Patient behavior immediately after transient ischemic attack according to clinical characteristics, perception of the event, and predicted risk of stroke. Stroke 37(5):1254–1260 10.1161/01.STR.0000217388.57851.62 16574923

[9] Mc Sharry J, Baxter A, Wallace LM (2014). Delay in seeking medical help following transient ischemic attack (TIA) or “mini-stroke”: a qualitative study. PLoS One.

[10] Chandratheva A, Lasserson DS, Geraghty OC (2010). Population-based study of behavior immediately after transient ischemic attack and minor stroke in 1000 consecutive patients: lessons for public education. Stroke.

[11] Wilson AD, Coleby D, Taub NA (2014). Delay between symptom onset and clinic attendance following TIA and minor stroke: the BEATS study. Age Ageing.

[12] Lasserson DS, Chandratheva A, Giles MF (2008). Influence of general practice opening hours on delay in seeking medical attention after transient ischaemic attack (TIA) and minor stroke: prospective population based study. BMJ.

[13] Hurst K, Lee R, Sideso E (2016). Delays in the presentation to stroke services of patients with transient ischaemic attack and minor stroke. Br J Surg.

[14] Geffner D, Soriano C, Perez T (2012). Delay in seeking treatment by patients with stroke: who decides, where they go, and how long it takes. Clin Neurol Neurosurg.

[15] Faiz KW, Sundseth A, Thommessen B (2013). Prehospital delay in acute stroke and TIA. Emerg Med J.

[16] Bruins Slot MH, Rutten FH, van der Heijden GJ (2012). Gender differences in pre-hospital time delay and symptom presentation in patients suspected of acute coronary syndrome in primary care. Fam Pract.

[17] Flynn D, Ford GA, Rodgers H (2014). A time series evaluation of the FAST National Stroke Awareness Campaign in England. PLoS One.

[18] Dombrowski SU, Mackintosh JE, Sniehotta FF (2013). The impact of the UK ‘Act FAST’ stroke awareness campaign: content analysis of patients, witness and primary care clinicians’ perceptions. BMC Public Health.

